# In Situ Testing of Polymers Immersed in Aging Fluids at Elevated Temperature and Pressure

**DOI:** 10.3390/ma15072690

**Published:** 2022-04-06

**Authors:** Bo Xu, Mark Redmond, Ahmed Hammami, Pierre Mertiny

**Affiliations:** 1 CAEVIS Technology Ltd., 31 Kingslake Rd., Toronto, ON M2J 3E2, Canada; bo.xu@caevis.com; 2 Shawcor Ltd., Corporate Research & Development, 25 Bethridge Rd., Toronto, ON M9W 1M7, Canada; mredmond@shawcor.com; 3 Shawcor Ltd., Composite Systems, 3501 54 Ave SE, Calgary, AB T2C 0A9, Canada; ahmed.hammami@shawcor.com; 4 Department of Mechanical Engineering, University of Alberta, 9211-116 St., Edmonton, AB T6G 1H9, Canada

**Keywords:** in situ material testing, polymers, punch-shear test, material aging and degradation

## Abstract

A novel elevated-temperature and high-pressure in situ punch-shear-test cell was developed to qualify materials for reliable service in harsh environments representative of those typically encountered in oil and gas operations. The proposed modular and compact test device is an extension of the ASTM D 732 punch-shear method. Conventionally, materials are first exposed to harsh environments, then removed from the aging environment for mechanical testing. This practice can lead to the generation of unrealistic (often optimistic) mechanical properties. This is especially true in the case of materials for which fluid ingress is reversible. The present contribution elaborates on the developed in situ punch-shear device that has been successfully used to realistically assess the tensile yield strength and modulus properties of in-service polymer materials based on experimentally established correlations between shear and tensile tests.

## 1. Introduction

Polymer materials and related composites are increasingly gaining attention and usage in many engineering applications related to the aerospace, automotive, and oil and gas sectors, to name a few. Compared to metallic engineering materials, polymer components offer ease of manufacture and selective resistance to a variety of chemicals (most notably corrosion resistance) while meeting specific stiffness and strength requirements. However, previous research has shown that properties of polymer materials can vary drastically between their original dry state and after aging in certain fluids [1,2,3,4]. Degradation mechanisms include plasticization, leaching, and chain scission. Especially for applications entailing prolonged fluid exposure with high pressure and/or elevated temperature, the use of such organic materials requires systematic pre-screening at realistic conditions for intended applications. It is imperative to assess long-term fluid compatibility to ensure safety and intended performance before placing polymer components into service.

In order to assess whether a selected polymer material is suitable for a given application, it is necessary to evaluate its mechanical properties at the intended operating conditions, including temperature, pressure, and fluid exposure to saturation. Conventional testing approaches involve exposing material samples to the desired environment until saturation, then removing the samples from the aging environment for mechanical testing. This practice can generate misleading results, especially for materials for which fluid ingress is rapidly reversible, most notably at elevated temperatures. For example, Yuan and Goodson [3] reported a “moisture-induced reversible process” in epoxy specimens, i.e., significant losses in tensile strength were largely recovered after a drying process.

Unfortunately, a widely accepted, commercially available and easy-to-use in situ test apparatus is not available to date. In fact, information in the technical literature on suitable testing equipment is scarce. In [2], a stand-alone high-temperature and high-pressure in situ environmental–mechanical test rig was proposed, providing tensile-, compression- and shear-test capabilities. However, compression testing indicated that the mechanical properties of the studied polymer were significantly different from results obtained with a conventional test method. The patent in [5] describes an in situ environmental–mechanical test apparatus with a tensile-test mode, yet no test results from this system have been reported. In [6], a specially designed environmental tensile-testing chamber was used to study the performance of fiber-reinforced plastics for offshore processing environments. Other specialized equipment has been developed for testing biomaterials [7] and examining material microstructures under in situ mechanical loading [8,9].

In the present paper, a new modular in situ testing apparatus and process are described. Based on the ASTM D732 punch-shear method [10], this apparatus is intended for studying the effects on mechanical properties when a material sample is in direct fluid contact at a defined temperature and pressure. The test system is expedient to operate because of its compact design and reduced risks in terms of health, safety and environment, especially when using hazardous fluids. The apparatus is actuated by a standard universal testing machine in compression mode. The validity of the test device and method was demonstrated by a pilot test series, which confirms an equivalence to conventional tensile testing according to ASTM D638 [11] and the ability to capture aging effects on material mechanical properties.

## 2. Experimental Setup

### 2.1. Test Apparatus

**Figure 1:**
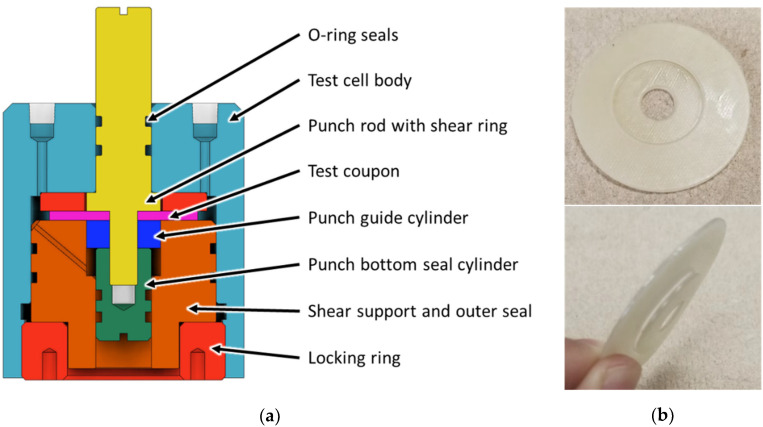
(**a**) Schematic of the in situ punch-shear-test cell, and (**b**) photographs of coupon sample after testing.

The in situ punch-shear-test cell depicted in Figure 1a enables measuring the shear properties of materials by performing punch-shear tests on coupons immersed in a fluid of interest at a desired temperature and pressure. For ease of manufacturing and operation, the setup employs 50.8 mm (2″) diameter round coupons as defined in the test standard ASTM D732 [10]. Figure 1b shows a coupon sample after testing.

While major components of the test cell correspond to ASTM D732, important differences include sealing mechanisms applied to contain the test fluid, and ports for charging and discharging the system with fluid. The test cell was designed to structurally sustain a pressure and temperature of 24 MPa and 250 °C, respectively. However, recognizing the limitations in the employed seals, the unit employed in this study was validated only to 10.5 MPa and 82 °C. The current test cell is made of stainless steel, yet for highly corrosive fluids such as hydrogen sulfide, more corrosion-resistant materials need to be used, such as the nickel alloy Hastelloy. As depicted in Figure 1a, the test coupon (pink color) is centered by the punch-rod core and secured between the top clamp spacer ring and the bottom shear support. The testing fluid fills the cavities in the test cell. The total fluid volume in this cell amounts to 12.5 mL. The test coupon is sheared when a load is applied at the top of the punch rod. The test is completed when the bottom shear ring of the punch rod enters the pocket of the bottom shear support. The load force and the displacement are recorded during the test through the universal testing machine. In this study, an Instron 5982 machine was used (Norwood, MA, USA).

**Figure 2:**
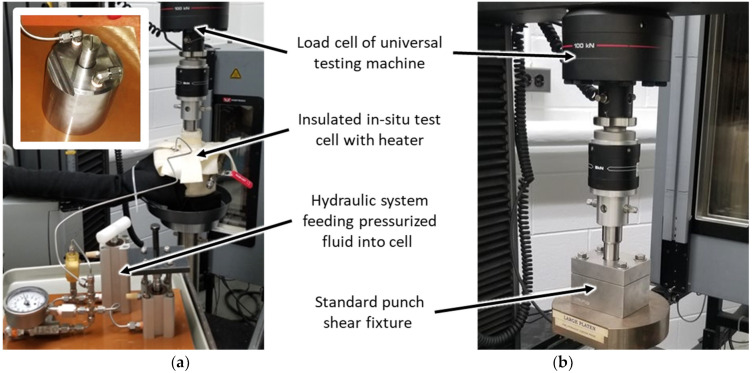
Photographs showing operation of (**a**) in situ test cell (inset: test cell without heater and insulation), and (**b**) standard ASTM D732 fixture.

The photographs in Figure 2 show the operation of (a) the in situ test cell and (b) a standard ASTM D732 punch-shear fixture (Wyoming Test Fixtures, Salt Lake City, UT, USA). For the in situ test cell, two compact hydraulic cylinders feed pressurized test fluid into the cell while the cell temperature is maintained by an external copper-coil heater circulating hot fluid. The outside of the heater and test cell is insulated with aramid felt insulation. The cell was designed to allow fluid to circulate isothermally and isobarically through an internal pressure-balancing port (Figure 1a, sloping port) within the shear support, allowing movement of the punch rod free of forces due to hydrostatic pressure, and a return flow path for incompressible fluids. The standard punch-shear fixture was utilized to validate the measurements obtained from the in situ test cell for dry samples at room temperature.

### 2.2. Materials and Sample Fabrication

Five different materials were tested in this study (see Table 1). At this juncture, it shall be emphasized that the objective herein was to assess the validity of the developed in situ punch-shear-test method rather than studying specific polymers, even though the selected materials have relevance to the authors’ work in the field of piping and vessels for oil and gas applications. Polymer A1 is generic high-density polyethylene (HDPE, specification ASTM D4976, obtained from McMaster Carr, Elmhurst, IL, USA). Polymers A2, B1, B2 and C are polyethylene with raised temperature capability, two maleic-anhydride-modified polyolefins, and an aliphatic polyketone, respectively. Note that the exact specifications for these materials are proprietary as they are used in industrial research and development work.

Circular punch-shear coupons with 50.8 mm (2”) diameter were prepared using a die cutter. The center hole in the test coupons was prepared with a 9.5 mm drill bit. Sheets of Polymer A1 with thicknesses of 0.8 mm, 1.6 mm, 2.3 mm and 4.8 mm were used to validate the in situ test-fixture design by comparing test results with those obtained from the standard ASTM D732 punch-shear fixture. Note that samples of Polymer A1 were not subjected to aging. For all other testing, including the study of various aging conditions, compression molding was employed to achieve a thickness of 2.0 mm for coupon samples of Polymers A2, B1, B2 and C. To establish a correlation between the results from punch-shear testing and conventional tensile testing, specimens of Polymer A2 were also prepared by conforming to ASTM D638 type I.

### 2.3. Conditioning in Aging Environments

Table 1 indicates the environments (i.e., fluid type, temperature, and pressure condition) to which the various materials were exposed, i.e., air, an aromatic test fluid, CO_2_ (100% purity gas from a pressure bottle with pressure booster system), and deionized water. Coupons made from Polymer A2 were exposed to the aromatic fluid for one month and then transferred to the in situ test fixture. The experiments were conducted after further exposure in the test cell for at least 8 h. Polymers B1, B2 and C were exposed to CO_2_ or deionized water in the in situ test cell for at least 48 h prior to testing.

### 2.4. Test Procedure and Data Reduction

Referring to ASTM D732, testing with both the standard punch-shear fixture and the in situ punch-shear-test cell occurred with a crosshead speed of the testing machine of 1.25 mm/min. Shear stress was calculated by dividing the force applied during the shearing of the specimen by the area of the sheared edge. The latter is determined by the product of the initial specimen thickness and punch-shear circumference. Since the diameter of the punch is constant (i.e., 25.4 mm), a simplified shear-stress calculation can be derived as,

(1) $$\tau =\frac{F}{25.4\pi t}=0.012532\frac{F}{t}$$

where *τ* is the shear stress in MPa, *F* is the applied force in N, and *t* is the coupon thickness in mm.

**Figure 3:**
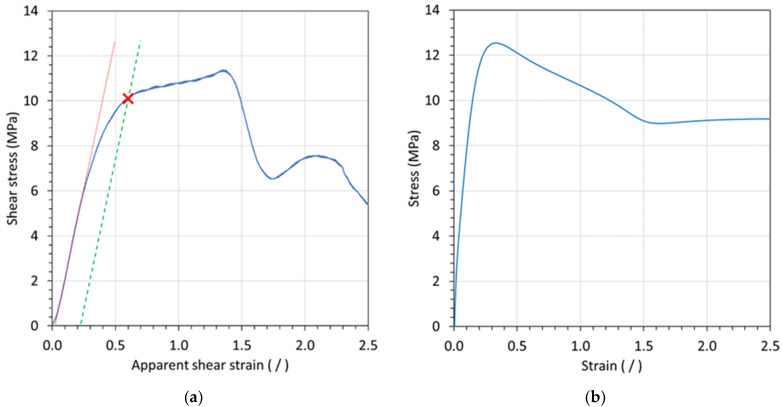
Typical stress–strain curve for (**a**) a punch-shear test and (**b**) a conventional tensile test using as ASTM D638 type I specimen (Polymer A2 tested in air at 60 °C).

Typically, only shear strength is reported when performing tests according to ASTM D732 (‘strength’ is defined based on maximum recorded load). However, it is desirable to collect more comprehensive information when testing aged specimens, due to the costs associated with long-term exposure and safety requirements when handling certain chemicals. In this work, the additional parameter ‘apparent shear strain’ was introduced to establish a stress-strain curve for punch-shear tests. The apparent shear strain is defined by dividing the shear displacement by the coupon thickness. Then, the apparent shear modulus is defined by the slope of the initial linear portion of the stress-strain curve, as shown by the red line in Figure 3a depicting a graph with a typical stress-strain curve for an in situ punch-shear test (for Polymer A2 tested in air at 60 °C). As shown by this graph, stress initially increased sharply, followed by a gradual drop in slope accompanied by rising strain prior to reaching the maximum stress. After the maximum stress, the curve drops sharply, followed by some stress oscillations with increasing strain. Notably, up to the maximum stress, the curve obtained by punch-shear testing resembles the stress-strain behavior of a conventional tensile test as depicted in Figure 3b for an ASTM D638 type I specimen under the same material and temperature conditions. Accordingly, a ‘shear strength at yield’ can be defined from the punch-shear test in order to provide an essential parameter for material evaluation and comparison. Akin to the offset-yield-point method for tensile testing (often set to 0.2%), the shear strength at yield was herein determined using an offset point that was taken as the stress value at which 20% apparent shear plastic deformation occurred (i.e., the intersection between the stress-strain curve and the parallel 0.2-strain offset of the modulus line as indicated by the green dashed line in Figure 3a). An apparent shear strain offset of 0.2 was herein adopted as it provided consistent results for the materials tested in this study.

## 3. Results and Discussion

### 3.1. Comparison of Test Results between Standard Punch-Shear Fixture and In Situ Test Cell

**Figure 4:**
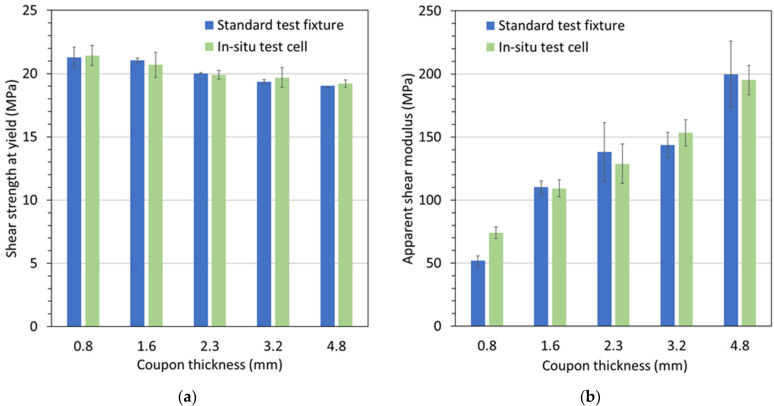
Comparison of (**a**) shear strengths at yield and (**b**) apparent shear moduli, obtained from testing with the standard punch-shear fixture and the in situ punch-shear-test cell, for coupons with different thicknesses made of Polymer A1.

A concern with the developed in situ punch-shear-test cell is the friction effects that can increase the measured force readings used in the shear-stress calculation. As mentioned earlier, the standard ASTM D732 punch-shear fixture was adopted for the in situ punch-shear-test cell, with an important modification being the seals for fluid containment. By carefully defining the dimension and tolerances for the punch rod, shear ring and shear-support pocket, and selecting proper O-ring seals and lubrication, the effect of friction between seals and sealing surfaces was minimized to an equivalent shear stress of less than 0.1 MPa, which was found to be within the experimental standard deviation. Therefore, results from the designed in situ test cell and the standard ASTM D732 punch-shear fixture should be comparable, which is confirmed by the graphs in Figure 4. Figure 4a depicts shear strengths at yield obtained from the two test fixtures for Polymer A1 and different coupon thicknesses. It can be seen from this graph that for each coupon thickness, the strength values are practically identical. Notably, with increasing coupon thickness, a slight drop in strength values can be observed, i.e., by ~10% from 0.8 mm to 4.8 mm coupon thickness. Note that all experiments presented in this study were completed in triplicate or greater.

In a similar fashion, apparent shear moduli are presented in Figure 4b. Again, for each coupon thickness, the results are in good agreement, except for the 0.8 mm coupon thickness. This discrepancy may be attributed to a rising geometric sensitivity relating to the gap tolerance between the shear ring and the shear-support-pocket cylinder, as well as the corner sharpness of these two components. Referring to ASTM D732, the recommended specimen thickness is between 1.27 mm and 12.7 mm because of inferior test results for specimens below the lower limit. Clearly, the present results corroborate the recommendation. Notably, a significant rise in apparent shear moduli was ascertained with increasing coupon thickness, i.e., an increase by ~80% from 1.6 mm to 4.8 mm coupon thickness. It is postulated that this effect arises from different effective shear rates for the different coupon thicknesses, given that the same rate of axial displacement was applied for all tests. In addition, bending effects across the gap between the shear ring and the shear-support cylinder may be responsible for an apparent shear-modulus reduction with diminishing coupon thickness. These results indicate that for comparative studies between materials and/or aging conditions, coupon thickness and loading rate should be kept constant.

### 3.2. Correlation between Data from Punch-Shear Testing and Tensile Testing

**Figure 5:**
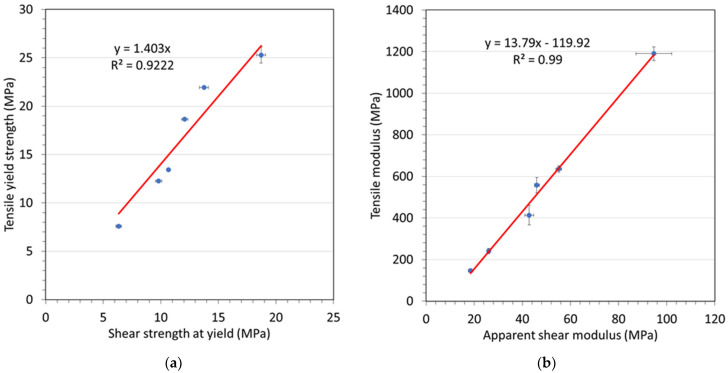
Scatter plots of (**a**) tensile yield strength versus shear strength at yield, and (**b**) tensile modulus versus apparent shear modulus, obtained from tensile and punch-shear testing, for coupons made of Polymer A2.

Material tensile properties are commonly used in product design and engineering evaluation. However, as alluded to earlier, the simultaneously tensile testing of samples and exposure to aging fluids is challenging, and related testing equipment is costly, potentially hazardous to operate, and not readily available. Therefore, correlating results from punch-shear testing to tensile properties is an attractive proposition, especially considering the capabilities of the developed in situ punch-shear-test cell. In recent research [13], data collected from punch-shear testing with an Sn-5Sb alloy were used to predict tensile properties as observed from direct tensile testing. Similarly, in the present study, data were collected from punch-shear testing and tensile testing for Polymer A2 at temperatures ranging from 23 °C to 82 °C (23 °C, 30 °C, 40 °C, 50 °C, 60 °C, 82 °C). These data were used to assess and corroborate a correlation between data from punch-shear and tensile testing. Accordingly, Figure 5 depicts scatter plots of (a) tensile yield strength versus shear strength at yield, and (b) tensile modulus versus apparent shear modulus. The graphs indicate that linear relationships exist between punch-shear- and tensile-test data. For the yield-strength data of the tested material, the relationship is as follows:

(2) $$\sigma_{y}=1.403\tau_{y}$$

where *σ_y_* is the tensile yield strength and *τ_y_* is the shear strength at yield. Similarly, the following expression is found for the modulus:

(3) $$E=13.79\;G_{S}-119.9=\alpha G_{S}-E_{0}$$

where *E* is the tensile modulus, *G*_S_ is the apparent shear modulus, and *E*_0_ is a constant.

The coefficients of determination (*R*^2^) associated with the datasets graphed in Figure 5 indicate satisfactory agreement with the regression lines (shown in red). Consequently, a correlation between punch-shear- and tensile-test data can be established, proposing that punch-shear testing can be used for predicting tensile performance once the correlation coefficients have been determined. Nevertheless, further testing is required to confirm this proposition using a broader set of polymer materials.

### 3.3. Effects of Exposure to Aging Fluids Observed via In Situ Punch-Shear Testing

**Figure 6:**
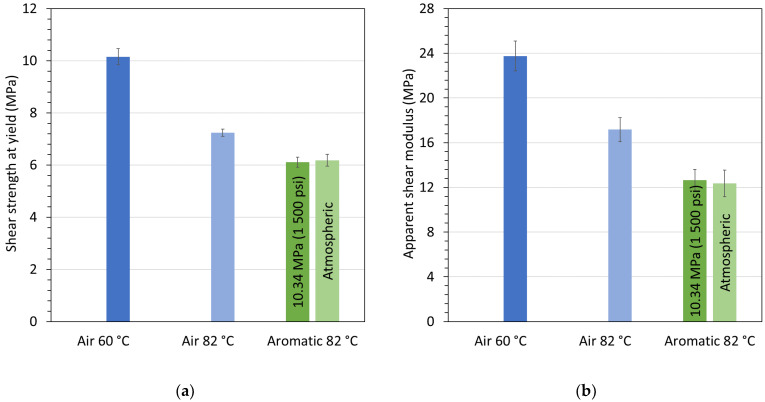
(**a**) Shear strength at yield and (**b**) apparent modulus for Polymer A2 conditioned and tested in air at 60 °C and 82 °C and in aromatic fluid at 82 °C and atmospheric and 10.34 MPa (1500 psi) pressure.

The final part of this text concerns the pilot test series that was conducted to demonstrate the validity and efficacy of the developed test cell. The effects of exposing the various materials to the aging fluids as listed Table 1 are presented in the following. First, the results from in situ punch-shear testing in terms of strength and modulus are shown in Figure 6a,b, respectively, for Polymer A2 conditioned and tested in air at 60 °C and 82 °C and in aromatic fluid at 82 °C and atmospheric and 10.34 MPa (1500 psi) pressure. Figure 6 reveals a significant decrease in shear strength and shear modulus from the low to the high temperature, with even greater reductions compared to room temperature, i.e., 45% and 70%, respectively (as indicated by the maximum values in Figure 5). The properties further deteriorated when the material was exposed to the aromatic fluid, yet there was no apparent difference in terms of fluid pressure.

**Figure 7:**
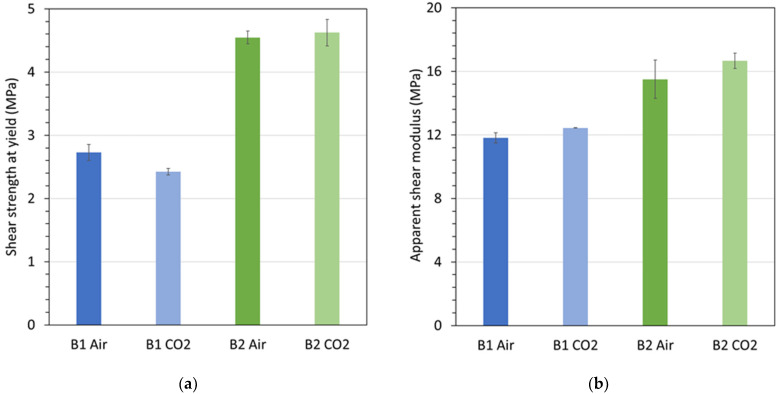
(**a**) Shear strength at yield and (**b**) apparent modulus for Polymers B1 and B2, each conditioned and tested at 82 °C in air and in CO_2_ at atmospheric and 6.89 MPa (1000 psi) pressure.

The graphs in Figure 7 depict results for (a) the shear strength at yield and (b) the apparent shear modulus, for Polymers B1 and B2 tested at 82 °C in air at atmospheric pressure and in CO_2_ gas at 6.89 MPa (1000 psi) pressure. Figure 7a indicates a reduction in shear strength for Polymer B1 when exposed to CO_2_ rather than air. In contrast, no such reduction was observed for Polymer B2. For both materials, the apparent shear modulus was not appreciably affected by CO_2_ exposure. It is hypothesized that the shear strength reduction for Polymer B1 in a CO_2_ environment is caused by the material absorbing sufficient amounts of CO_2_ that led to weakening.

**Figure 8:**
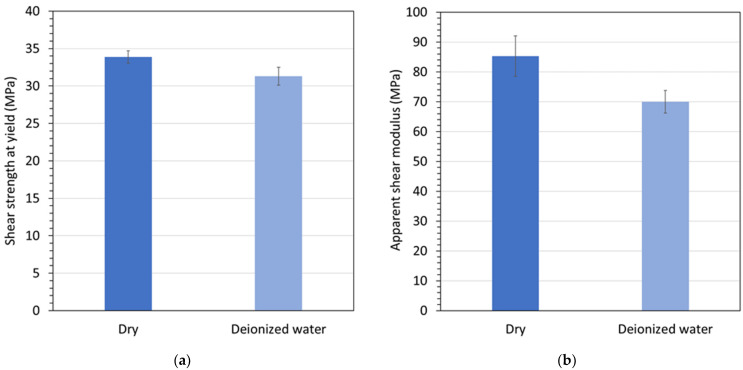
(**a**) Shear strength at yield and (**b**) apparent modulus for Polymer C conditioned and tested at 82 °C in air and in deionized water.

Finally, Figure 8 demonstrates the hygroscopic effects of Polymer C at 82 °C. The graphs in Figure 8 indicate that both the shear strength at yield and the apparent shear modulus were reduced by water exposure and saturation, likely due to a water-induced plasticizing effect that is reversible with drying [14], as discussed in the following.

**Figure 9:**
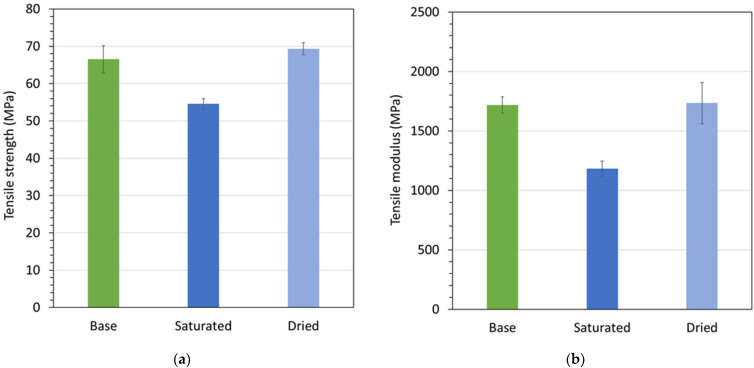
(**a**) Tensile yield strength and (**b**) modulus at room temperature for Polymer C pre-aging (base) and post-aging (saturated and dried) in deionized water at 105 °C and 150 kPa for 10 days.

To underline the importance of in situ testing, the graphs in Figure 9 are presented. Figure 9a,b correspondingly depict tensile yield strengths and moduli at room temperature for Polymer C at a pre-aging state and post-aging conditions in deionized water at 105 °C and 150 kPa. The post-aging conditions comprise samples that were tested immediately after 10 days of aging and samples that were subsequently left to dry at 100 °C in vacuum for 24 h. The data in the figure show that hygroscopic aging caused a substantial drop in strength (18%) and modulus (31%), yet the loss in performance was recoverable when the aging fluid was allowed to desorb from the material. These results clearly demonstrate the viability of the developed in situ punch-shear-test cell. Polymers readily desorb previously absorbed fluid vapor, especially for highly volatile fluids and/or at temperatures approaching the fluid boiling point. Consequently, conventional testing of fluid-aged samples in dry atmosphere is prone to produce results that fail to capture the full exposure effects.

## 4. Conclusions

A novel, modular in situ punch-shear-test device was developed for measuring the mechanical strength of polymers under realistic service conditions of temperature, pressure and fluid saturation. A series of experiments was performed to explore the validity and effectiveness of this fixture, employing several thermoplastic polymer materials and aging conditions. It was shown that results obtained with the in situ test cell agree reasonably well with room-temperature-test data that were produced using a conventional ASTM D732 punch-shear fixture. The experiments suggest that material properties, i.e., yield strength and modulus, derived from punch-shear testing can be correlated to conventional tensile-testing results, which would provide opportunities for generating properties for engineering analysis and design in an expedient manner. However, this supposition requires further validation. Finally, the efficacy of the in situ punch-shear-test cell for generating material data for comparative studies was made evident through testing polymer samples at elevated temperatures and exposure to fluids under volatile conditions. In future work, it is anticipated to employ the in situ test cell for the testing of polymers under severe and hazardous fluid exposure.

## Figures and Tables

**Table 1:** Materials tested for aging effects in specified environments.

Material	Aging Environment		
	Fluid	Temperature (°C)	Pressure (MPa; psi)
Polymer A2	Air	60	Atmospheric
	Air	82	Atmospheric
	Aromatic ^1^	82	Atmospheric
	Aromatic ^1^	82	10.34; 1500
Polymer B1	Air	82	Atmospheric
	CO_2_	82	6.89; 1000
Polymer B2	Air	82	Atmospheric
	CO_2_	82	6.89; 1000
Polymer C	Air	82	Atmospheric
	Deionized water	82	Atmospheric

^1^ Aromatic fluid composition: 50% IRM 902 (ASTM D5964 [12]), 8.33% cyclohexane, 8.33% xylene, 8.34% toluene, 15% octane, 6% heptane, 4% hexane.

## Data Availability

Not applicable.
